# Genome-wide identification and comprehensive analysis of *NAC* family genes involved in fruit development in kiwifruit (*Actinidia*)

**DOI:** 10.1186/s12870-020-02798-2

**Published:** 2021-01-15

**Authors:** Dongfeng Jia, Zhiqiang Jiang, Haihui Fu, Lu Chen, Guanglian Liao, Yanqun He, Chunhui Huang, Xiaobiao Xu

**Affiliations:** 1 College of Agronomy, Jiangxi Agricultural University, Nanchang, 330045 Jiangxi China; 2 Institute of Kiwifruit, Jiangxi Agricultural University, Nanchang, 330045 Jiangxi China

**Keywords:** Kiwifruit, NAC transcription factor, *Actinidia eriantha*, Fruit development, Gene expression

## Abstract

**Background:**

NAC transcription factors (TFs) are plant-specific proteins encoded by a large gene family. They play important roles in diverse biological processes, such as plant growth and development, leaf senescence, and responses to biotic or abiotic stresses. Functions of a number of NAC TFs have been identified mainly in model plants. However, very few studies on NAC TFs have been conducted in the fruit tree of kiwifruit.

**Results:**

Genome-wide *NAC* genes were identified and their phylogeny, genomic structure, chromosomal location, synteny relationships, protein properties and conserved motifs were analyzed. In addition, the fruit developmental process was evaluated in a new kiwifruit cultivar of *Actinidia eriantha* ‘Ganlu 1’. And expressions for all those *NAC* genes were analyzed by quantitative real-time PCR method in fruits of ‘Ganlu 1’ during its developmental process. Our research identified 142 NAC TFs which could be phylogenetically divided into 23 protein subfamilies. The genomic structures of those *NAC* genes indicated that their exons were between one and ten. Analysis of chromosomal locations suggested that 116 out of 142 *NAC*s distributed on all the 29 kiwifruit chromosomes. In addition, genome-wide gene expression analysis showed that expressions of 125 out of 142 *NAC* genes could be detected in fruit samples.

**Conclusion:**

Our comprehensive study provides novel information on *NAC* genes and expression patterns in kiwifruit fruit. This research would be helpful for future functional identification of *NAC* genes involved in kiwifruit fruit development.

## Background

Kiwifruit belongs to *Actinidia*, which is a large genus containing more than 50 species, and China is the original center of kiwifruit [1]. Fruit of kiwifruit is called “the king of fruits” as its remarkably high vitamin C content and rich nutritional minerals for human health. By domestication and selection from wild resources, many kiwifruit varieties have been developed, so kiwifruit has become one of the most widely cultivated fruit trees around the world [2–4]. *Actinidia* species are dioecious plants, and their ovary from female flower is formed by several carpels. Their fresh berry fruit usually contains a lot of seeds [5], which is helpful for successfully reproducing under complex environments. The development of kiwifruit berry fruits starts from fertilization and ends until seed maturation [6]. It was reported that during the fruit development of *A. chinensis* ‘Hort16A’, many aspects of fruit morphology, growth and development are similar to that of tomato (*Solanum lycopersicum*) fruit, except for the delay production of autocatalytic ethylene in the ripening process when fruit begins to senesce [6, 7].

In most cases, gene structures can be combined with expression analysis to give an insight into gene function prediction. Usually, genes with close structures have similar functions, and identifying gene family is a useful way for functional test of genes. However, so far, very few gene families have been identified in the kiwifruit genome. Notably, the genome sequence of *A. chinensis* ‘Hongyang’ is available since the completion of its genome project [3]. Transcription factors (TFs) are a class of proteins that regulate the temporal and/or spatial expressions of target genes by binding to *cis*-regulatory elements in their promoter regions [8]. Plant TFs, including AP2, bHLH, ARF, MYC, WRKY, and NAC, are essential regulators in many biological processes [9–13]. Among those TFs, NACs play important roles in diverse developmental processes in plants, such as plant growth, lateral root formation, leaf senescence, and fruit ripening and softening [14–17].

NAC protein family is one of the largest TF families in plants. A typical NAC protein contains a conserved NAC domain at its N-terminal, which comprises about 150 amino acids, and a highly variable transcriptional regulation region at the C-terminus [18, 19]. Since the first report of NAC protein in 1996 [20], NAC protein families have been identified in several plant species, such as *Arabidopsis thaliana* [19], rice [21], poplar [22], grapevine [23], and Asian pear [24]. In addition, functions of a number of *NAC* genes have been uncovered in model plants. Overexpression of *AtATAF1*, a *NAC* gene of *A. thaliana*, enhances plant tolerance to drought stress [25]. Overexpressing *OsNAC10* in rice improves plant drought stress tolerance and grain yield [26]. *PopNAC122* of poplar reduces plant height growth by reducing cell size and cell number [27]. In addition to regulatory functions in stress response and plant growth, NAC proteins also play important roles in regulating fruit development. In tomato, NAC TFs of SlNAC4 and NOR-like 1 have been identified as positive regulators of tomato fruit ripening [17, 28]. And tomato *SlNAC1* gene can alter fruit pigmentation and fruit softening in both ethylene-dependent and abscisic acid-dependent pathways [16]. In strawberry (*Fragaria chiloensis*), FcNAC1 protein involves in fruit softening by regulating pectin metabolism [29]. And in cucumber (*Cucumis sativus*), 12 *NAC* genes are identified to be targets of 13 micro-RNAs and they are involved in fruit development [30].

Although NAC TFs play potential roles during stress responses and developmental processes, the specific functions of most *NAC* genes are still poorly understood, especially for those genes in kiwifruit, an important kind of berry fruit tree. To date, no systemic analysis on *NAC* gene family has been conducted in kiwifruit. In this study, genome-wide identification of NAC proteins was conducted in the genome of *A. chinensis* ‘Hongyang’. And comprehensive studies on phylogeny, gene structure, chromosomal location, and protein properties were also performed. In addition, the developmental process of fruit was evaluated in a new kiwifruit cultivar of *A. eriantha* ‘Ganlu 1’. And relative expressions of all those identified *NAC* genes were investigated by quantitative real-time PCR (qRT-PCR) method in fruits of ‘Ganlu 1’ during its developmental stages. Our results would be valuable for cloning and functional identification of kiwifruit *NAC* genes. The expressing profiling data will provide essential information for revealing regulatory mechanisms of NAC TFs on kiwifruit fruit growth and development.

## Results

### Fruit development of *A. eriantha* ‘Ganlu 1’

**Fig. 1:**
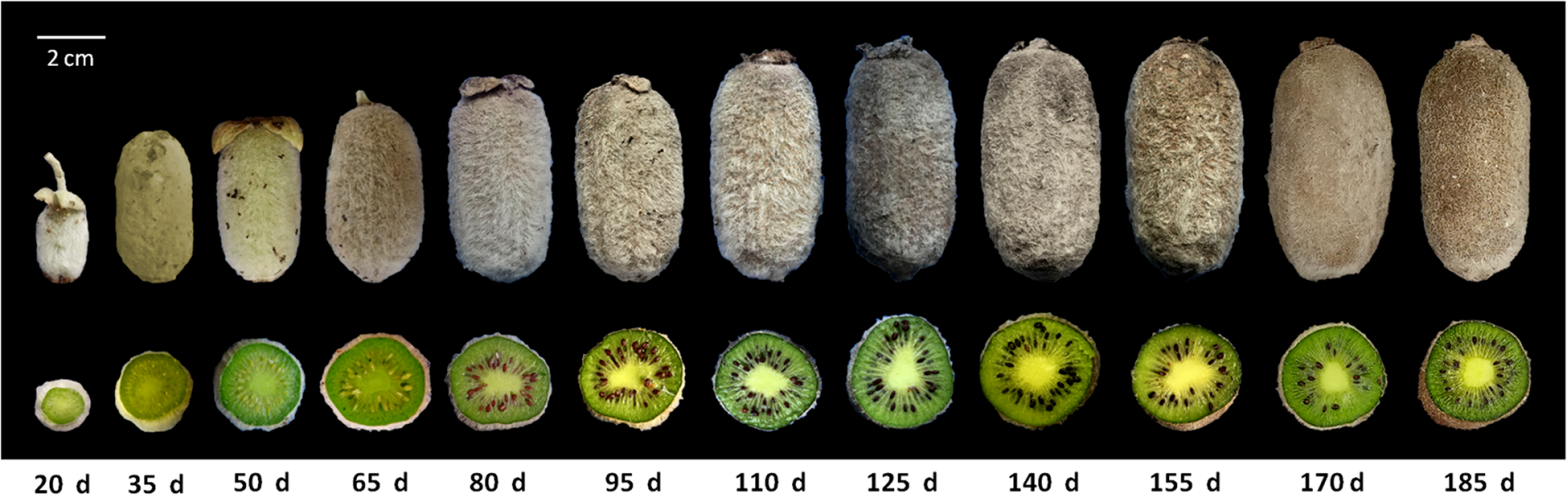
Fruits of *Actindia eriantha* ‘Ganlu 1’ collected at different developmental stages. d: day after flowering

As shown in Fig. 1, during the developmental process from the 20 day after flowering (DAF) to the 185 DAF of ‘Ganlu 1’ fruit, the fruit appearance changed obviously at different stages. Specifically, the fruit size increased rapidly during the early developmental stage (from 20 DAF to 35 DAF). And the seeds within outer pericarp were observed from the 50 DAF and from then on. In this process, the seed color was yellow (50 DAF), then turned to red brown (80 DAF), and then became black (110 DAF) (Fig. 1).

**Fig. 2:**
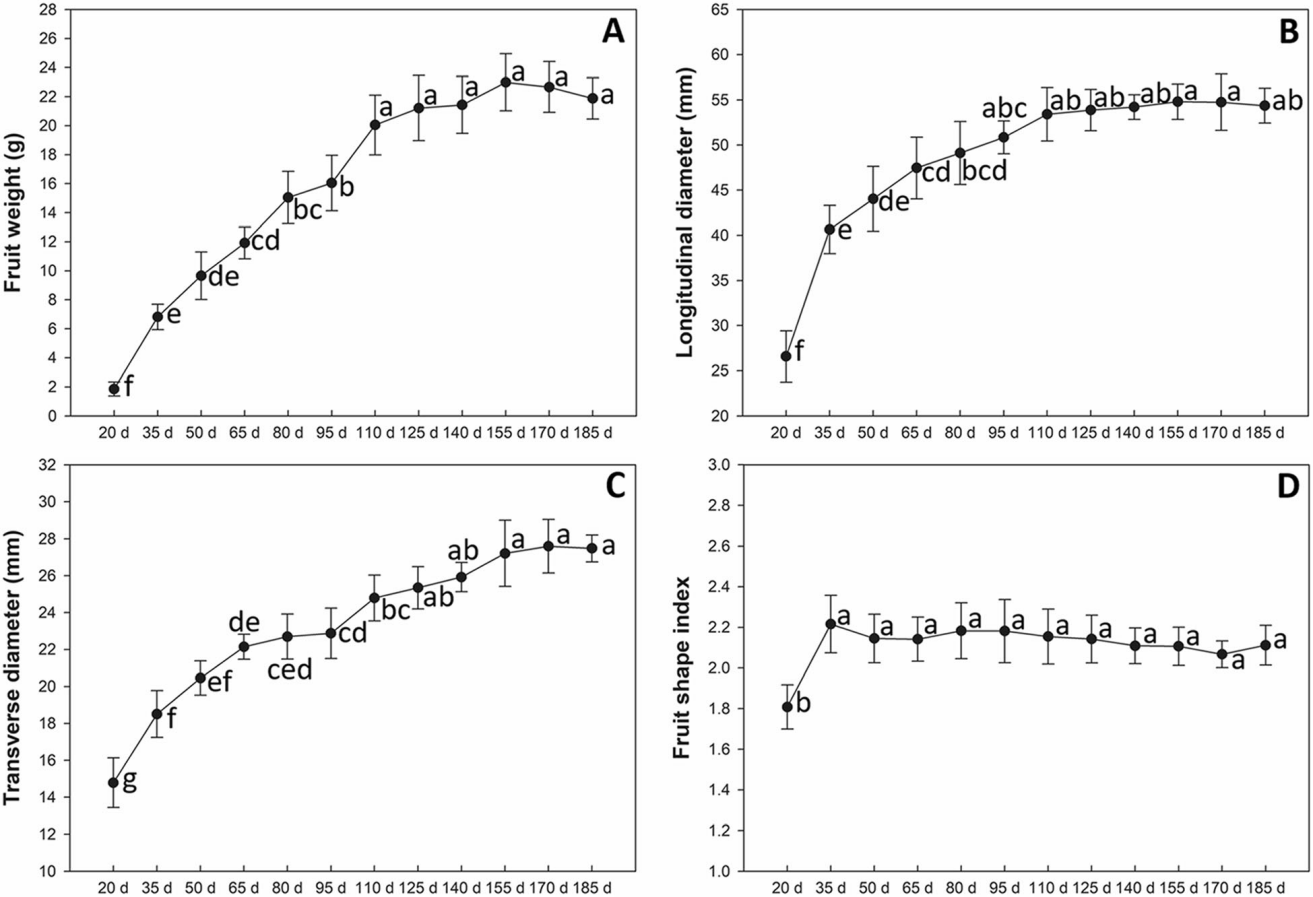
Longitudinal and transverse diameters of fruit during the developmental stages in *Actinidia eriantha* ‘Ganlu 1’. The values are presented as mean ± standard deviation. The data were assessed by one-way ANOV and Tukey’s multiple comparison tests (*p* < 0.05). The significant differences of the data are shown by lower case letters beside the bars. d: day after flowering

In addition, the fruit, longitudinal diameter, transverse diameter and fruit shape index of fruit were analyzed. The fruit weight increased rapidly during the developmental periods from 20 DAF to 110 DAF (Fig. 2a). The fruit longitudinal diameter increased rapidly during the early developmental periods (from 20 to 35 DAF), then slowly increased (from 35 to 110 DAF), and it tended to be stable in the late developmental periods (from 110 to 185 DAF) (Fig. 2b). The fruit transverse diameter showed a different variation trend than that for longitudinal diameter. The fruit exhibited a quick increasing trend for the transverse diameter during the earlier stages from the 20 to 65 DAF, and it exhibited a second growth peak during the 95 to the 125 DAF (Fig. 2c). However, the values of fruit shape index increased significantly only from 20 to 35 DAF, then they stayed the same until to 185 DAF (Fig. 2d).

### Kiwifruit NAC transcription factors and their sequence features

We identified a total of 142 *NAC* genes in the genome of *A. chinensis* ‘Hongyang’ and they were designated as *AcNAC001* to *AcNAC142*, following their identifiers from the Kiwifruit Genome Database (http://kiwifruitgenome.org/) (Additional file 1). Our analysis confirmed that each of these identified AcNAC proteins contained a NAM domain (PF02365.15), a specific conserved domain of NAC TF protein family (Additional file 2). In addition, these AcNAC proteins also showed sequence similarity with *Arabidopsis* NAC proteins (Additional file 3).

The coding sequence (CDS) lengths of these *AcNAC* genes were between 438 bp and 1962 bp. And these AcNAC proteins contained 145 to 653 amino acids in length, with an average number of 328 amino acids (Additional file 4). The molecular weights were from 15,926.35 to 74,381.42 Da, with an average value of 37,227.39 Da. The predicted isoelectric points of these NAC proteins were from 4.39 to 10.24 (Additional file 4).

### Phylogenetic relationships of NAC proteins

**Fig. 3:**
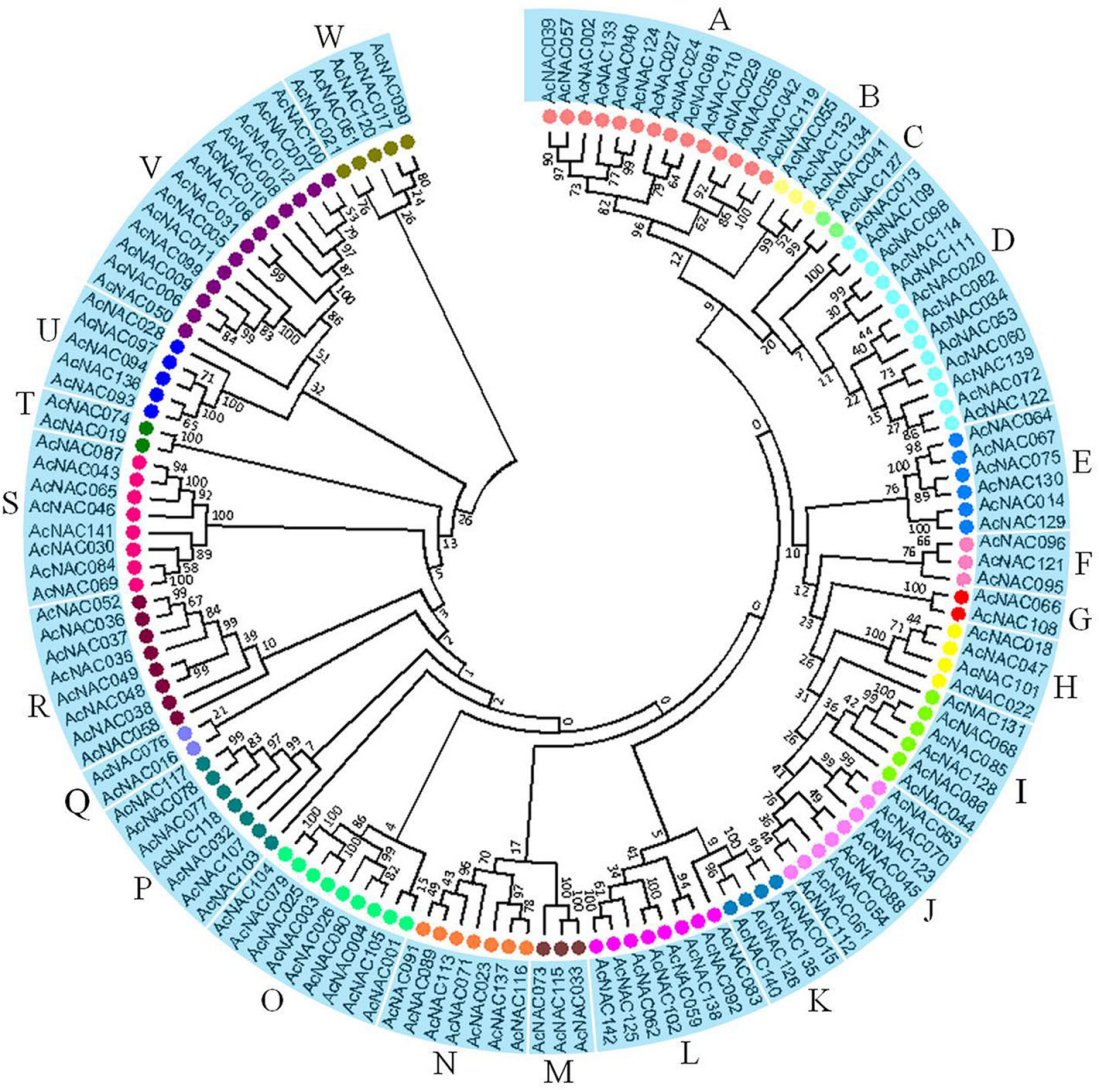
Phylogenetic tree of NAC proteins in kiwifruit. The phylogenetic tree was constructed by MEGA software (version 5.05) using neighbor-joining method, with 1000 bootstrap replicates. These 23 protein subfamilies are indicated by different colors, and their names are marked with upper case letters

The unrooted phylogenetic tree of AcNAC proteins allowed us to separate the kiwifruit NAC protein family into 23 subfamilies. For simplicity, those subfamilies were designated alphabetically as A to W (Fig. 3). Among these subfamilies, subfamily A included 14 AcNACs members, followed by 13 in subfamily D and V, 9 in O, 8 in J, L, R and S, 7 in N and P, 6 in E and I, 5 in U and W, 4 in H and K, 3 in B, F and M. However, subfamilies of C, G, Q and T each contained only two NAC members (Fig. 3).

In addition, to investigate the phylogenetic relationships of NAC proteins from kiwifruit and *Arabidopsis*, the phylogenetic tree was constructed using all those NAC protein sequences of kiwifruit as well as that of *Arabidopsis*. The phylogenetic tree showed that NAC proteins of kiwifruit and *Arabidopsis* could be divided into 26 subfamilies, which were designated as subfamily A to Z (Additional file 5). In most cases, the same subfamilies from both of the two phylogenetic trees (Fig. 3 and Additional file 5) contained identical members (such as subfamilies of A, C, E, F, G, H, I, J, K, L, M, N, O, Q, S, T, U and V). However, there were several exceptions, AcNAC111 from subfamily D in phylogenetic tree of kiwifruit AcNACs (Fig. 3) was clustered into subfamily B in phylogenetic tree constructed by NACs of kiwifruit and *Arabidopsis* (Additional file 5). The cases were similar for AcNAC058, AcNAC090, AcNAC103 and AcNAC139 (Fig. 3, Additional file 5). These above four NAC proteins along with corresponding *Arabidopsis* NACs formed X, Y, Z new subfamilies (Additional file 5). Besides, members of subfamilies F, I, R and V were all from kiwifruit; conversely, NAC members of the other 22 subfamilies were from both kiwifruit and *Arabidopsis* (Additional file 5). These results may indicate the special functions of NAC members within those four subfamilies in kiwifruit.

### Genomic lengths, gene structures and conserved protein motifs

**Fig. 4:**
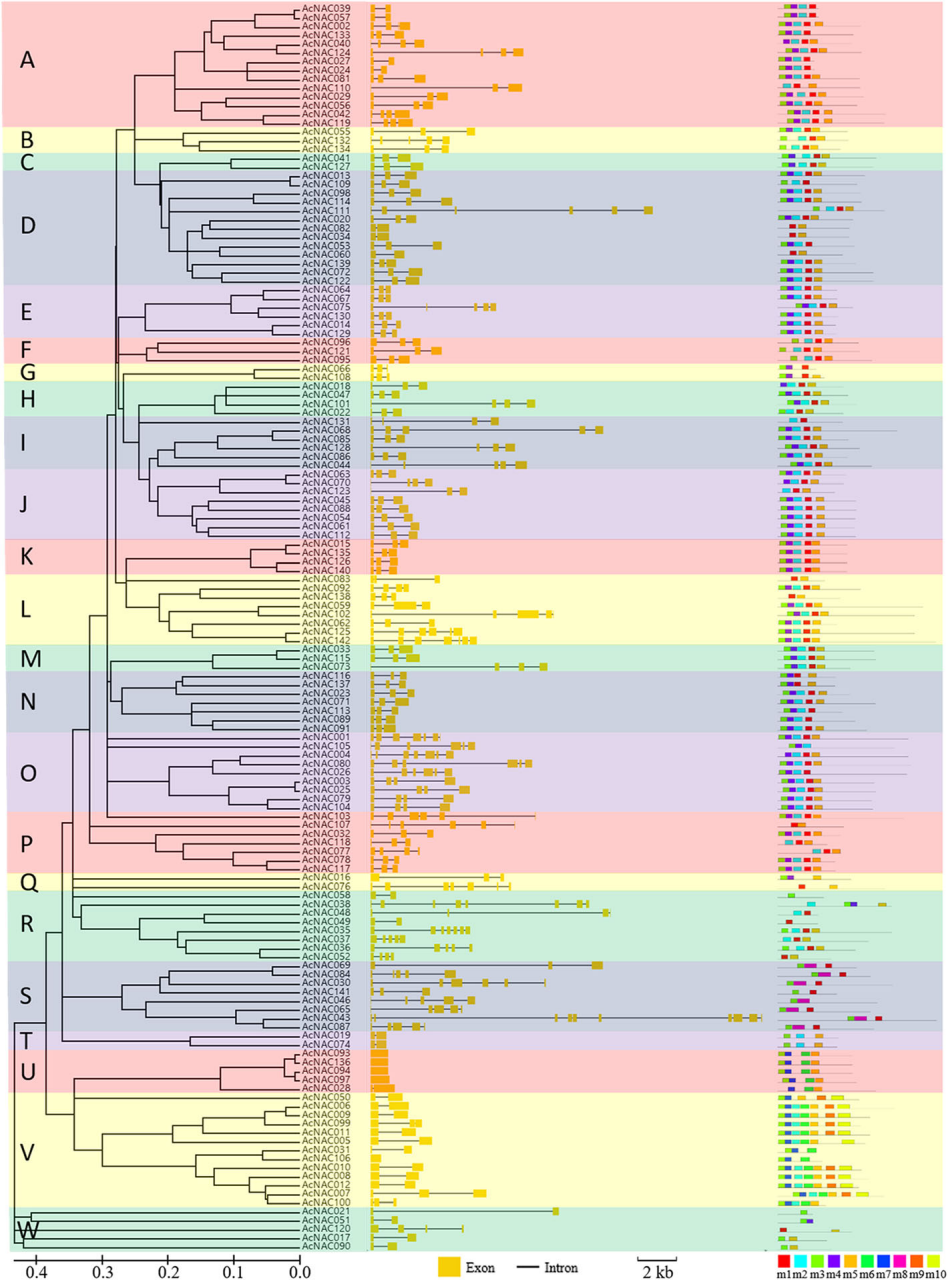
Genomic structures of *AcNAC* genes and conserved motifs of AcNAC proteins. The yellow block and black line on the right represent exon and intron, respectively (middle). These subfamilies of NAC proteins are indicated by different colorful blocks and their names are marked with upper case letters on the left (left). These blocks with different colors show the names of protein motifs (right)

Among these 142 *AcNAC* genes, the genomic structures were greatly different. For exon numbers, 67 *AcNAC*s contained 3 exons (47.2%), 25 contained 2 (14.6%), 19 contained 4 (13.4%), 11 contained 5 (7.7%), 8 contained 6 (5.6%), 5 contained 7 (3.5%), and the other 5 *NAC* genes (3.5%) of *AcNAC093*, *AcNAC136*, *AcNAC094*, *AcNAC097* and *AcNAC106* each had only one exon and did not contain any intron. Besides, *AcNAC038* contained 8 exons (0.7%), and *AcNA043* contained the most of 10 exons and 9 introns (0.7%) (Fig. 4, Additional file 6). For all these *AcNAC* genes, the average numbers of exon and intron were 3.4 and 2.4, respectively (Fig. 4).

The lengths of nucleic acid bases for these *AcNAC* genes also varied greatly, their genomic lengths were between 555 bp and 20,432 bp, with an average genomic length of 3592.3 bp (Fig. 4, Additional file 6).

For those 142 NAC proteins, 10 conserved motifs were detected by employing MEME models (Fig. 4, Additional file 7). All these NAC proteins contained at least one conserved motif. And these motifs distributed at the N-terminal for most of those NAC proteins. In general, those closely related NACs within the same subfamily had similar motif compositions. Within the AcNAC protein family, 120 out of the whole 142 members contain motif 3, indicting their conserved domains.

### Chromosomal locations and synteny relationships of *AcNAC* genes

**Fig. 5:**
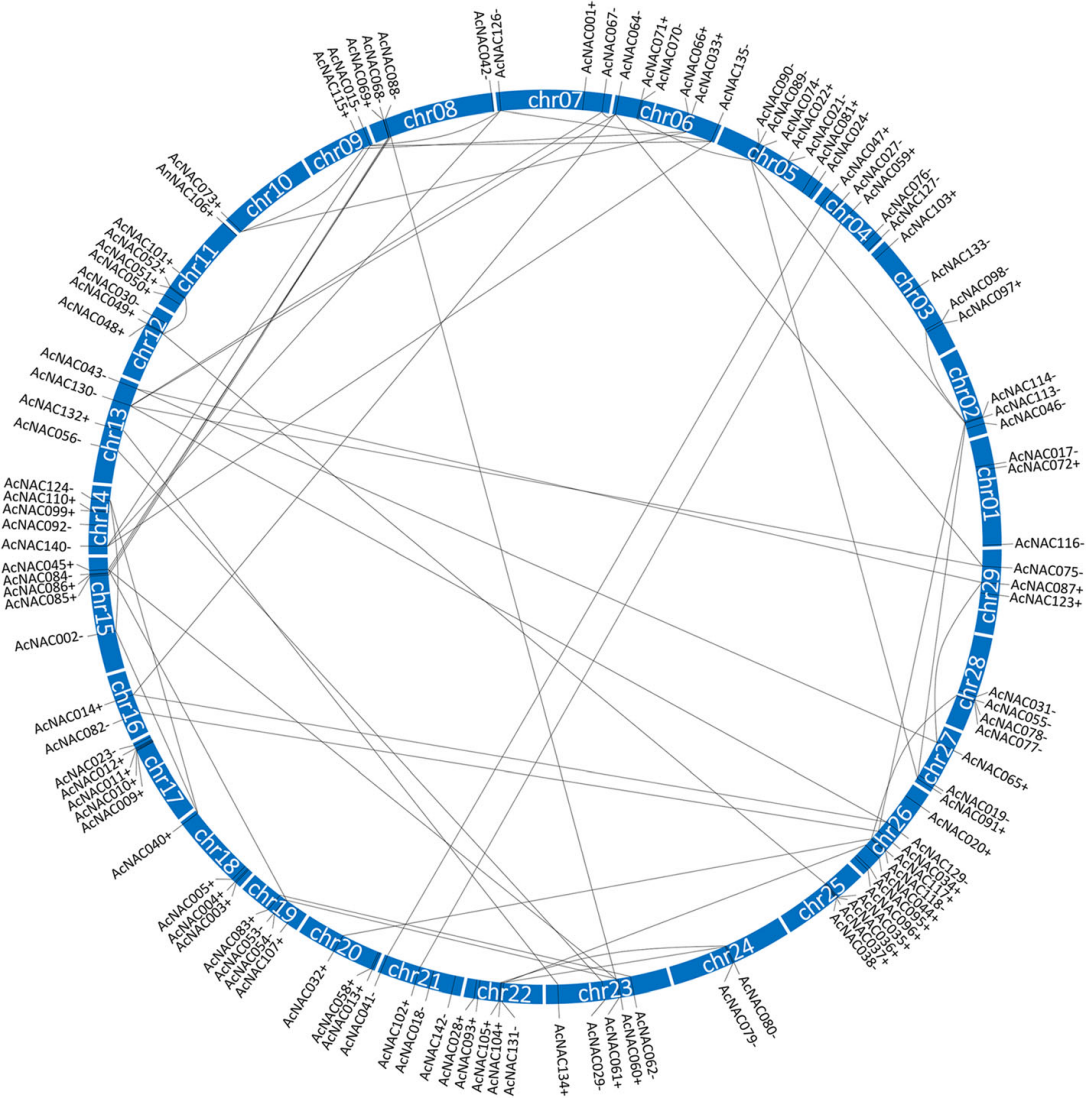
Chromosomal distributions and synteny relationships of *AcNAC* genes in the genome of *Actinidia chinensis*. The kiwifruit *NAC* genes are marked in the vertical directions of chromosomes. “+” and “-” represent the forward strand and reverse strand, respectively. The gene pairs with synteny relationships are linked by black lines

Chromosomal locations of 116 out of these 142 *AcNAC* genes was acquired from the Kiwifruit Genome Database (Additional file 8). The 116 *AcNAC*s were distributed along all the 29 kiwifruit chromosomes (chrs). The distribution was neither equal nor random. They were oriented either forward (+ strand) or in reverse (− strand). The numbers of *AcNAC* genes for each individual chr were from 1 (Chr10) to 8 (Chr26), and the average numbers of *AcNAC* genes on one chr were 4 (Fig. 5).

For distribution of *AcNAC* genes on these chromosomes, 62 out of the 116 *AcNAC* genes (53.4%) distributed on forward strands (+ strand), and 54 out of the 116 *AcNAC* genes (46.6%) distributed on reverse strands (− strand) (Fig. 5, Additional file 8). However, chromosomal location information of the rest 26 *AcNAC* genes could not be available from the Kiwifruit Genome Database.

In addition, according to the duplicated blocks in the *A. chinensis* ‘Hongyang’ genome, 49 gene pairs with synteny relationships were identified (Fig. 5), and their genomic block information was shown in Additional file 9.

### Expression patterns of *NAC* genes in fruit during its developmental process

**Fig. 6:**
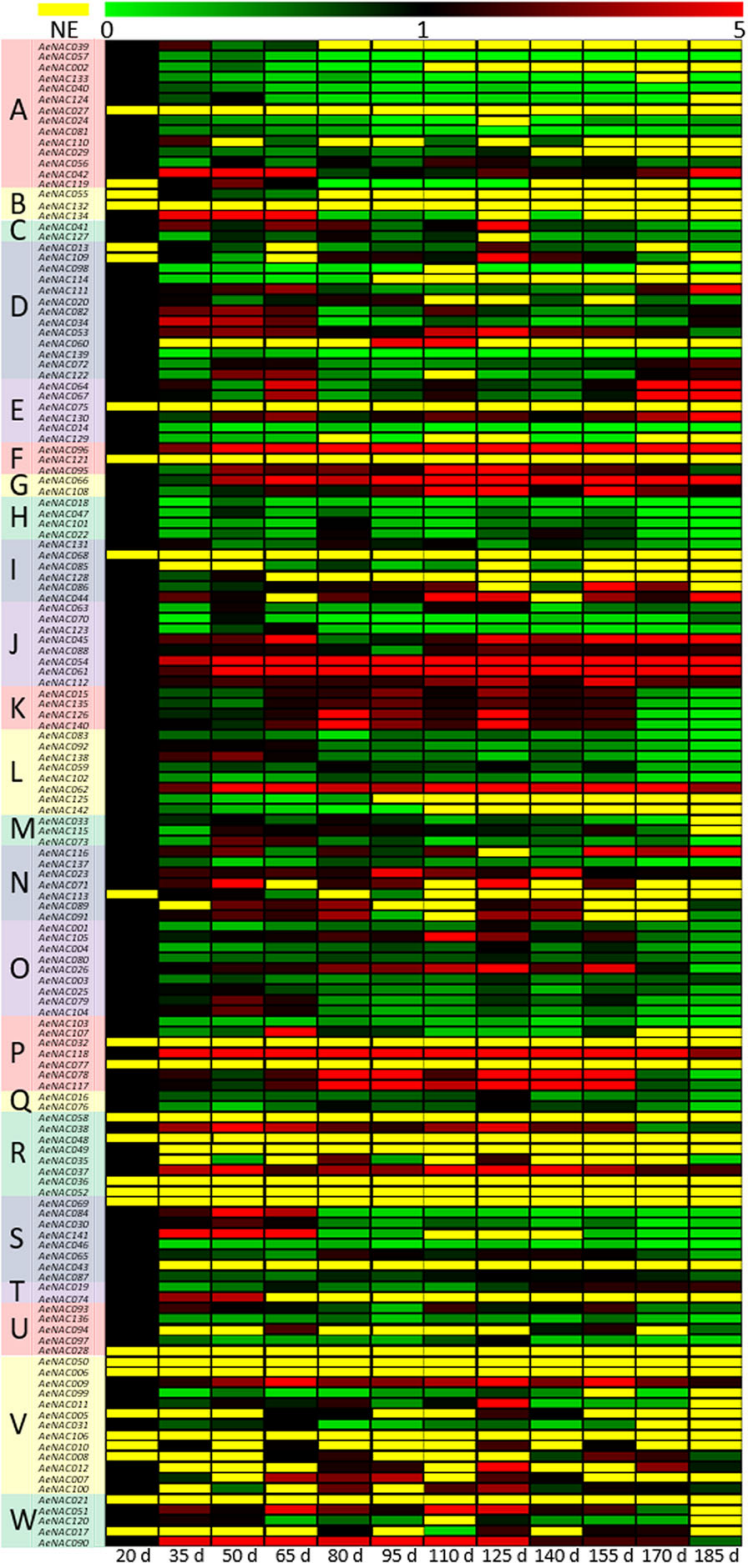
Genome-wide relative expression levels of *NAC* genes in fruit during the developmental process in *Actinidia eriantha* ‘Ganlu 1’. The blocks with different colors represent the relative expression levels comparing with the control. The yellow blocks represent no expression (NE) in the samples. d: day after flowering

The relative expression levels of all these 142 *NAC* genes were analyzed in fruit at different developmental stages by qRT-PCR method. The expression levels for corresponding genes of *AcNAC*s (*AcNAC001* to *AcNAC142*) in *A. eriantha* ‘Ganlu 1’ (*AeNAC001* to *AeNAC142*) were analyzed. The data of expression patterns of 142 *AeNAC* genes were shown in Additional file 10. Among these genes, 74 *NAC*s persistently expressed in fruit during the whole developmental process, and 51 *AeNAC*s expressed in partial fruit samples at certain developmental stages. However, expressions of 17 *AeNAC* genes were not detected at any stages in fruit of *A. eriantha* ‘Ganlu 1’ during the whole developmental process (Fig. 6, Additional file 10).

Among the 74 *AeNAC* genes expressed in all the fruit samples, the expression patterns were also greatly different. Compared to the control (20 DAF), 28 *AeNAC*s showed down-regulation patterns at some stages, and 9 *AeNAC*s showed up-regulation patterns at some stages. Besides, 28 *AeNAC*s showed either down- or up-regulation patterns at certain stages. In addition, 6 *AeNAC*s (*AeNAC037*, *AeNAC054*, *AeNAC061*, *AeNAC062*, *AeNAC096* and *AeNAC118*) exhibited higher expression levels in all the fruit samples during the developmental process comparing with the control. On the contrary, 4 *AeNAC*s (*AeNAC014*, *AeNAC046*, *AeNAC103* and *AeNAC139*) showed decreased expression levels at each stage comparing with the control. Interestingly, the expression level of only one *AeNAC* gene, *AeNAC087*, did not change during the whole fruit developmental process (Fig. 6, Additional file 10). Among these different subfamilies, all gene members from subfamily H (*AeNAC018*, *AeNAC047*, *AeNAC102*, and *AeNAC022*) showed a decreased expression trend, these results may indicate their similar and negative roles in fruit development. However, within subfamilies J or O, some members showed notable different expression features, this also suggested *NAC* genes with similar structures may function differently. In short, those positive or negative relations between *NAC* expressions and fruit development may suggest their possible roles in regulating fruit development in kiwifruit, although they may act in a complex and diverse manner.

**Fig. 7:**
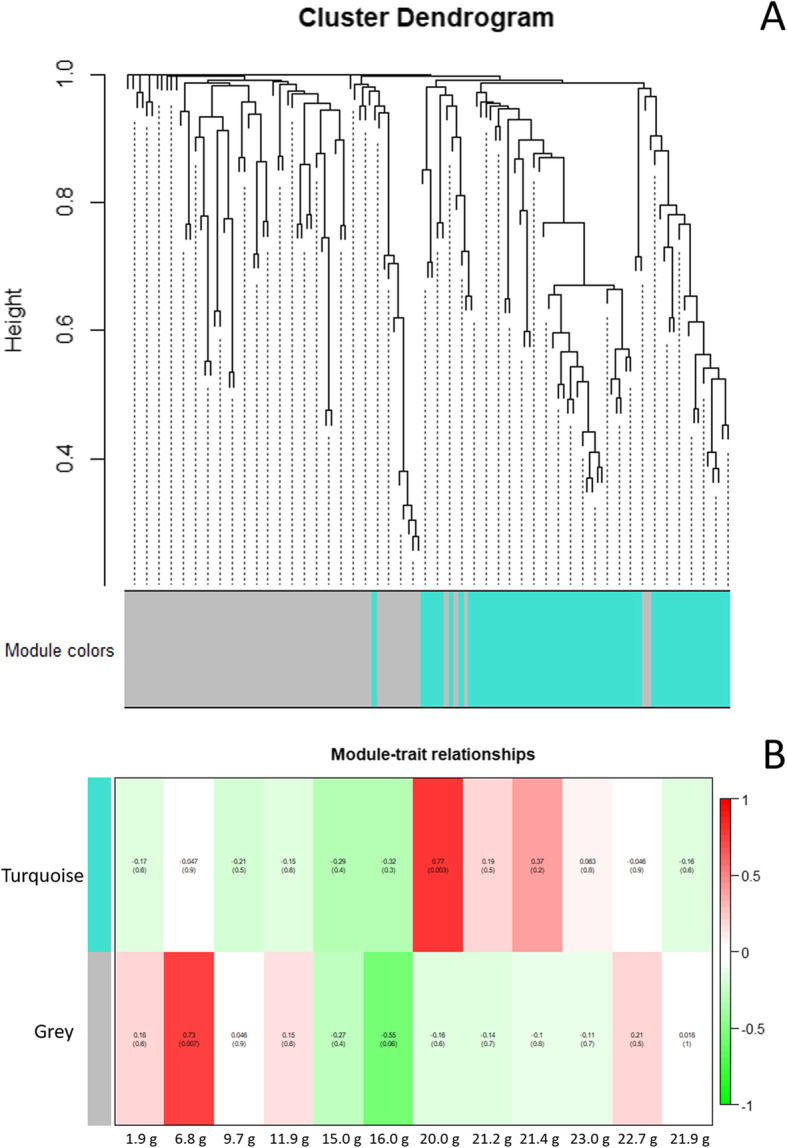
Hierarchical cluster tree with dissimilarity based on topological overlap showing coexpression modules identified by weighted gene co-expression network analysis (**a**). Each leaf in the tree shows one *AeNAC* gene. The branches constitute two modules labeled by turquoise and grey colors, respectively. **b**, Module-developmental stages association. The color of each block indicates the correlation coefficient between the module and the different developmental stages. The correlation coefficient R (upper) and *P*-value (lower) in each block are indicated by upper number and lower number, respectively

The weighted gene co-expression network analysis (WGCNA) showed that two modules (turquoise and grey, respectively) were identified in the 142 *AeNAC* genes (Fig. 7a). The module-trait relationships showed that the module turquoise was closed related with the middle and late fruit developmental stages, and the module grey was mainly related with the early developmental stages (Fig. 7b). In addition, the network relationships of the *NAC* genes identified in module turquoise and module grey were shown in Additional file 11 and Additional file 12, respectively.

## Discussion

In higher plants, fruit development is a sophisticated process, which is controlled by a number of biological and molecular mechanisms and is affected by environmental factors [31]. Understanding the regulatory pathways related to fruit development and ripening is very important for fruit quality improvement. Transcriptional regulation is an efficient way for controlling fruit development and ripening. Among those multiple TF genes, many *NAC* genes have been characterized to play essential roles in regulating fruit development. For example, in tomato, *SlNAC033* and *SlNAC064* control fruit development via modulating expressions of ethylene biosynthesis and cell wall metabolism related genes [17, 28]. Notably, little is known about the regulatory roles of *NAC* genes in kiwifruit fruit development.

NAC protein family is a large plant-specific TF family. Since the first report of NAC [20], many studies have been conducted on NAC families in different plant species. For example, 105 NAC proteins have been identified in *Arabidopsis* [19], 140 in rice [21], 163 in poplar [22], 74 in grapevine [23], and 185 in Asian pear [24]. The large number of *NAC* genes in various plant species indicates that they probably have extensively expanded in their evolutionary processes. To date, no systematic studies have been conducted on NAC family in the kiwifruit genome. Here, we identified a total of 142 kiwifruit NAC proteins based on its whole genome data. These NAC proteins were phylogenetically clustered into 23 subfamilies. Notably, NAC members of four subfamilies of F, I, R and V were all from kiwifruit (Additional file 5), suggesting that these genes may have a different ancestor with that of *Arabidopsis* during the evolutionary processes. Interestingly, the similar cases were also found in *NAC* gene family of tomato [32]. Hence, we speculated that those kiwifruit-specific *NAC* genes may have specialized roles in kiwifruit plant.

In the present study, each AcNAC protein contained a conserved NAM domain (PF02365.15), a specific feature of NAC TF proteins [18]. By analyzing the chromosomal locations of *AcNAC* genes, we found that 116 *AcNAC*s distributed on all the 29 kiwifruit chrs. Among them, some *AcNAC* genes showed “clustered” distribution patterns on certain chrs, such as, *AcNAC085*, *AcNAC086*, *AcNAC084* on Chr15, *AcNAC009*, *AcNAC010*, *AcNAC011*, *AcNAC012* on Chr17, and *AcNAC035*, *AcNAC036*, *AcNAC037*, *AcNAC038* on Chr25 (Fig. 5). Notably, most *NAC* genes within a same “gene clusters” belonged to the same subfamily, and they shared similar lengths and genomic structures, and the same conserved motifs (Fig. 3, Fig. 4). It is worth mentioning that in apple and tomato, many adjacent related genes (gene clusters) on the same chr also displayed similar structures. And they were reported to be produced by tandem duplication events as a result of genome-wide duplication [33, 34]. Similarly, in the evolutionary process of kiwifruit genome, distributions of NBS–LRR genes indicate that tandem duplication events also happened [3]. In addition, the large number of paralogous *NAC* gene pairs derived from genomic blocks indicated the segmentally duplicated events also happened in the genome evolution process. Therefore, we assume that the large number of *NAC* genes may be partially caused by the tandem duplication and segmental duplication events during the evolutionary process of kiwifruit genome, which may also have important impacts on the expanding of other genes in kiwifruit genome.

To confirm *NAC* genes are closed involved in fruit development, genome-wide expressions of all those 142 *NAC* genes were examined in the developmental fruit in the new kiwifruit cultivar of *A. eriantha* ‘Ganlu 1’. Particularly, three *NAC* genes, *AeNAC054*, *AeNAC061*, and *AeNAC118* showed constitutive high expression levels during the fruit development. In addition, these *NAC* genes were also identified as hub genes by using WGCNA. Overall, those positive correlations between fruit development and gene expressions of related *NAC* genes probably indicated their potential regulatory roles during kiwifruit fruit growth and development. Furthermore, some NAC proteins have been reported to be involved in fruit development as well as plant growth. SlNAC4 and NOR-like 1 were proved to be positive regulators of fruit ripening in tomato [17, 28]. In banana (*Musa acuminata*), four NAC proteins (MaNAC016, MaNAC083, MaNAC094, and MaNAC095) were proposed to function in pulp-ripening process based on gene expression analysis [35]. FcNAC1 participates fruit ripening development in strawberry (*Fragaria chiloensis*) via activating *FcPL*, which is involved in fruit cell wall remodeling [36]. *Arabidopsis NAC* genes of *AtNAP* and *JUB1* positively regulate fruit senescence and plant longevity [37, 38]. In addition, in woody plant apple (*Malus domestica*) and poplar (*Populus trichocarpa*), two *NAC* genes, *MdNAC1* and *PopNAC122*, were reported to reduce plant growth in overexpression transgenic plants [27, 39]. Nevertheless, more genetic evidence is required for further understanding the functions of *NAC* genes in kiwifruit growth and development.

## Conclusion

In this study, genome-wide identification and expression analysis of *NAC* genes in the kiwifruit genome were performed for the first time. Although some *NAC* genes in other plants have been reported to be widely involved in regulating plant growth and stress responses [40, 41], the study on *NAC* genes in kiwifruit is still in its infancy. Our work provided crucial candidate *NAC* genes involved in fruit growth and development. Our study also has practical value for developing early-maturing kiwifruit varieties to meet market requirements. However, further investigations still should be done to clarify the precise mechanisms of related *NAC* genes in regulation of fruit growth and development in kiwifruit, as well as in other fruit trees.

## Methods

### Plant materials and fruit development

Fruit trees of *A. eriantha* ‘Ganlu 1’ were planted at the Germplasm Resources Nursery of Institute of Kiwifruit, Fengxin County (28°70′ N, 115°38′ E), Jiangxi Province, China. Fruit samples were collected on the 20, 35, 50, 65, 80, 95, 110, 125, 140, 155, 170 and 185 DAF, respectively, during the developmental process (Fig. 1). The outer pericarp without seeds was cut into sections and rapidly frozen in liquid nitrogen, and stored at − 80 °C until use.

To evaluate fruit size during the developmental process, longitudinal diameter and transverse diameter of fruits collected at each stage were measured. At least 8 fruits were measured for each experiment. The data were assessed by one-way ANOV and Tukey’s multiple comparison tests (*p* < 0.05) using SPSS Statistics software (version 20).

### Genome-wide identification of NAC protein family

Genome sequence data of *A. chinensis* ‘Hongyang’ were downloaded from the Kiwifruit Genome Database [3] and they were used for identifying members of NAC TF protein family. Firstly, we performed a local BLASTP program search by BioEdit software [42] against all these genome protein sequences of kiwifruit, using 105 *Arabidopsis* NAC protein sequences (Additional file 3) downloaded from the *Arabidopsis* Information Resource site (https://www.arabidopsis.org/) as queries with an expectation value (E-value) < 1.0. Secondly, the predicted NAC protein sequences of *A. chinensis* were also downloaded from the Plant Transcription Factor Database (http://planttfdb.cbi.pku.edu.cn/) to collect entire candidates of NAC proteins in kiwifruit. All candidates collected by the above two methods were used for further identification. Three steps were taken to verify all these candidates. First, hidden Markov models (HMMs) of the Pfam 32.0 database (http://pfam.xfam.org/) were used to identify the NAM domain (PF02365.15) for each protein sequence under an E-value < 1.0 [43]. Second, conserved domains of the National Center for Biotechnology Information (https://blast.ncbi.nlm.nih.gov/) were also exploited to confirm the existence of NAM domains for all identified sequences [44]. Those protein sequences that do not contain NAM domain or have ambiguous domain belonging to other protein family were excluded. Third, we carefully examined all these candidate sequences and manually excluded protein sequences with a length less than 130 amino acids. Finally, these remaining protein sequences were regarded as NAC TFs of *A. chinensis* (AcNACs) and they were used for subsequent evaluation.

LaserGene software (version 7.1) was used to analyze the predicted isoeletric point and molecular weight of AcNAC proteins. And DNAMAN software (version 6.0) was used for the multiple sequence alignment analysis of AcNAC proteins and their corresponding similar sequences from *Arabidopsis*.

### Phylogenetic analysis of kiwifruit NAC proteins

To analyze the phylogenetic relationships of the AcNAC proteins, multiple sequence alignments were performed among these AcNAC proteins using MUSCLE method with default parameters [45]. An unrooted phylogenetic tree was constructed by MEGA software (version 5.05) using neighbor-joining method, with 1000 bootstrap replicates [46]. These same methods were also used for constructing phylogenetic tree of NAC proteins from kiwifruit and *Arabidopsis*.

### Analysis of structures and conserved motifs of proteins

Information on genomic chromosomal location and CDS of each *AcNAC* gene was downloaded from the Kiwifruit Genome Database. The genomic length and introns/exons organization for each individual *AcNAC* gene were displayed by the Gene Structure Display Server (version 2.0) (http://gsds.cbi.pku.edu.cn/) [47]. Details of genomic length and exons for those *AcNAC* genes (*AcNAC001* to *AcNAC142*) were presented in Additional file 6.

The conserved motifs in those 142 AcNAC protein sequences were elucidated using online MEME tool (version 5.1.1; http://meme-suite.org/). Zero or One Occurrence Per Sequence was used and motif width was set between 19 and 50 residues.

### Chromosomal distribution analysis and gene synteny analyses

The relative locations of *AcNAC* genes on different chrs as well as their chromosomal directions (+ strand or - strand) were also analyzed. The detailed information was shown in Additional file 8.

To explore the synteny relationships of *NAC* genes in the genome of *A. chinensis* ‘Hongyang’, the information of paired homologous *NAC* genes with common genomic blocks was obtained from the Kiwifruit Genome Database and was used to show the synteny relationships between *NAC* genes. The detailed information was shown in Additional file 9.

### Genome-wide expression analysis of *NAC* genes in developmental fruit and their gene co-expression network analysis

Total RNA of frozen fruit samples was extracted using a RNA isolation Kit (Huayueyang, Beijing, China) with a step of removing genomic DNA by DNase I. First-strand cDNA was synthesized with a PrimeScript Reverse Transcriptase Kit (Takara; Dalian, China).

To clarify the expression patterns of *NAC* gene in developmental fruits of *A. eriantha* ‘Ganlu 1’, the relative expressions of all those 142 *AeNAC* genes were analyzed by qRT-PCR method using a LightCycler 480 Real-Time PCR machine (Applied Biosystems, Waltham, MA, USA). The qRT-PCR analysis was performed with specific primers, using *AcNAC* sequences as templates (Additional file 13). The results were calculated with 2^-ΔΔCT^ method [48]. The kiwifruit *Actin* gene (GenBank accession number: FG515334.1) (Additional file 13) was used as the internal control to standardize the cDNA samples. Each experiment was conducted with three replicates.

To visually describe the expressing results, compared with the control (the first day when expression detected, mostly the 20 DAF), those relative expression levels with a fold change > 2.0 or < 0.5 were designated as increasing or decreasing expression, respectively. Meanwhile, when fold change of relative expression levels were between 0.5 and 2.0, those expressing results were defined as unchanged.

In addition, to identify the correlations among all those *AeNAC* genes, WGCNA was conducted using the average relative expression data (Additional file 10) of the 142 *NAC* genes with an R package [49]. The modules and their corresponding gene network data were obtained according to previous method [44] and those data were referred to identify hub genes. The module-developmental traits association analysis was also conducted [50]. The key R script parameters (optimum beta value and co-expression matrix) used for WGCNA were shown in Additional file 14.

## Supplementary Information

**Additional file 1. ***AcNAC* genes and their predicted coding or protein sequences identified in the genome of *Actinidia chinensis* ‘Hongyang’.

**Additional file 2.** AcNAC proteins and their conserved NAM domains identified by hidden Markov models.

**Additional file 3. **Similarity of kiwifruit NAC proteins and the corresponding NAC proteins from *Arabidopsis*.

**Additional file 4.** Primary information of AcNAC proteins and the lengths of their corresponding coding sequences (CDS).

**Additional file 5. **Phylogenetic tree of NAC proteins from kiwifruit and *Arabidopsis*. The phylogenetic tree was constructed by MEGA software (version 5.05) using neighbor-joining method, with 1000 bootstrap replicates. Subfamilies of NAC proteins are indicated by different colors, and their names are marked with upper case letters.

**Additional file 6. **Genomic length, exon number and length of *AcNAC* genes.

**Additional file 7.** Amino acid residue sequences of conserved motifs identified in the AcNAC proteins.

**Additional file 8. **Chromosomal locations and distribution directions of *AcNAC* genes.

**Additional file 9. **Synteny relationships and their genomic blocks of *AcNAC* gene pairs.

**Additional file 10. **Relative expression data of 142 *NAC* genes in fruit during the developmental process in *Actinidia eriantha* ‘Ganlu 1’.

**Additional file 11.** The enriched cluster information of expressing comparisons for the input genes from turquoise module identified by weighted gene co-expression network analysis.

**Additional file 12.** The enriched cluster information of expressing comparisons for the input genes from grey module identified by weighted gene co-expression network analysis.

**Additional file 13 **Primer sequences used for relative expression analysis of *AeNAC* genes with quantitative real-time PCR methods.

**Additional file 14.** The key R script parameters of optimum beta value and co-expression matrix used for weighted gene co-expression network analysis.

## Data Availability

The datasets supporting the conclusions of this article are available online from the Kiwifruit Genome Database, the Arabidopsis Information Resource site, the GenBank database, or included within the article additional files.

## Abbreviations

CDS: Coding sequence
chr: Chromosome
HMM: Hidden Markov models
NE: No expression
qRT-PCR: Quantitative real-time PCR
TF: Transcription factor
WGCNA: Weighted gene co-expression network analysis
