# Supplementary figures and images for: Genome-wide identification and comprehensive analysis of NAC family genes involved in fruit development in kiwifruit (Actinidia)
Source: BMC Plant Biol. 2021 Jan 15;21:44. doi: 10.1186/s12870-020-02798-2 (PMC7811246; doi:10.1186/s12870-020-02798-2)

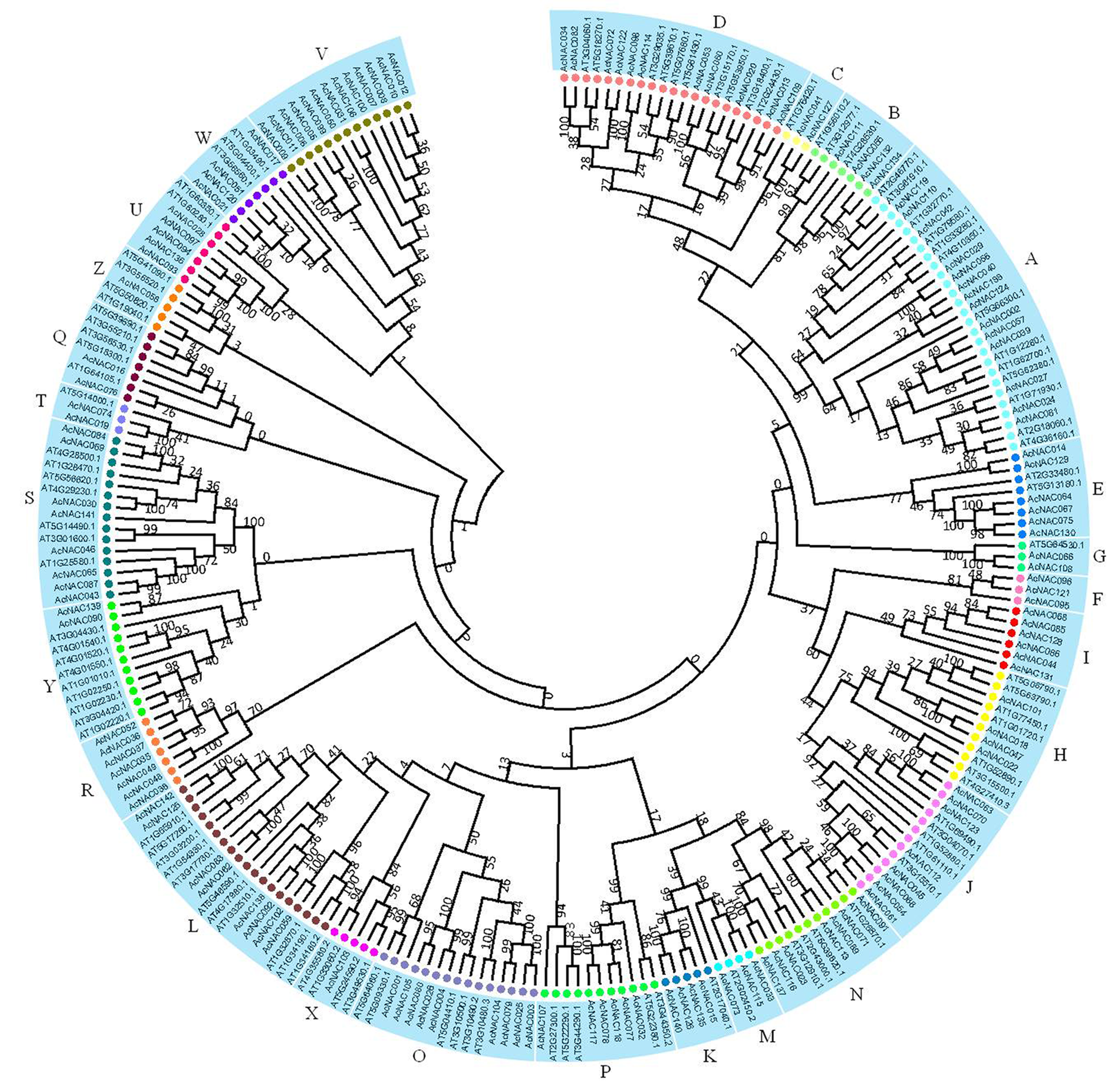

Supplement: Supplementary file 5 — Additional file 5. Phylogenetic tree of NAC proteins from kiwifruit and Arabidopsis. The phylogenetic tree was constructed by MEGA software (version 5.05) using neighbor-joining method, with 1000 bootstrap replicates. Subfamilies of NAC proteins are indicated by different colors, and their names are marked with upper case letters. [file 12870_2020_2798_MOESM5_ESM.tif]

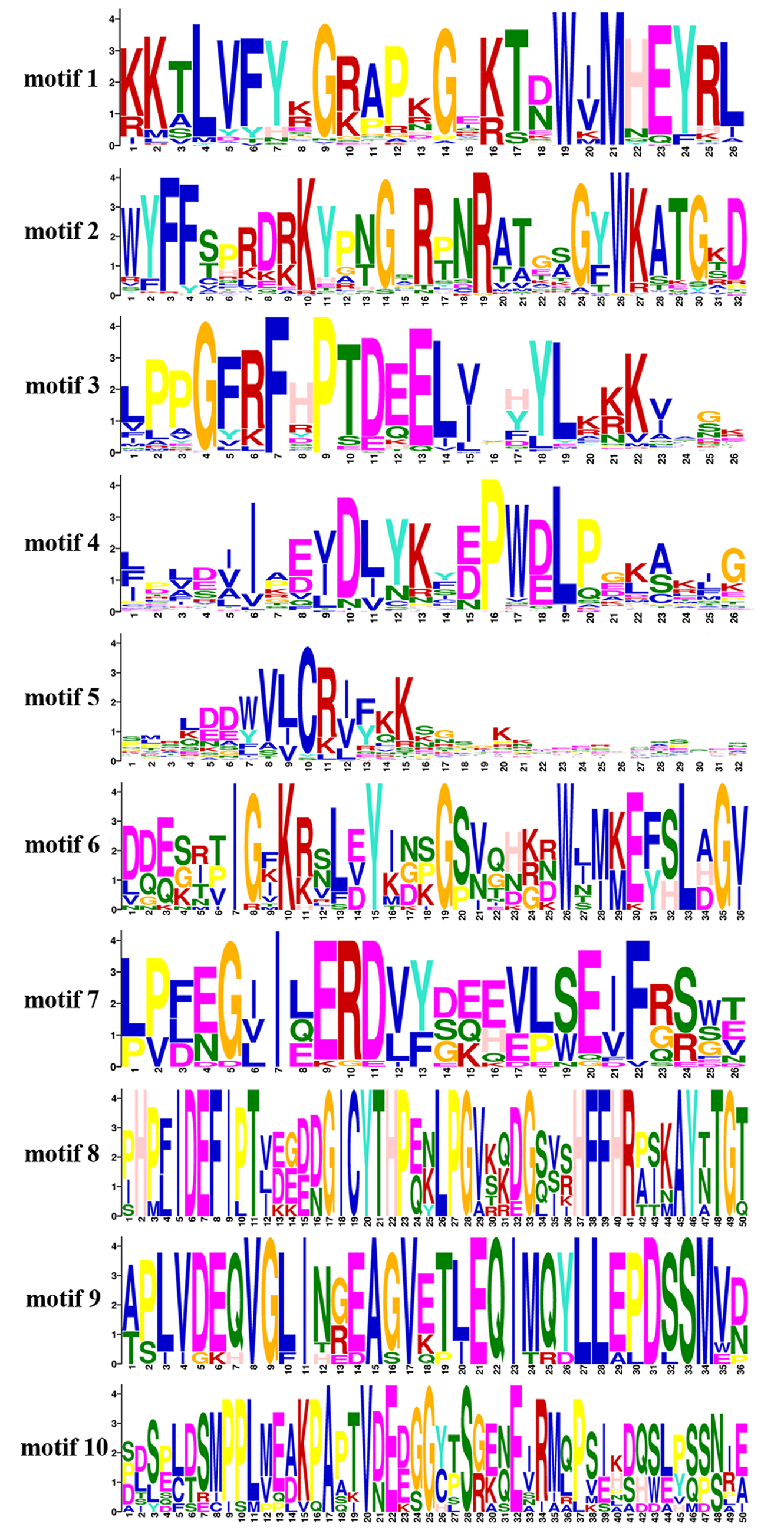

Supplement: Supplementary file 7 — Additional file 7. Amino acid residue sequences of conserved motifs identified in the AcNAC proteins. [file 12870_2020_2798_MOESM7_ESM.tif]
