# Supplementary material for: Genome-wide identification and comprehensive analysis of NAC family genes involved in fruit development in kiwifruit (Actinidia)
Source: BMC Plant Biol. 2021 Jan 15;21:44. doi: 10.1186/s12870-020-02798-2 (PMC7811246; doi:10.1186/s12870-020-02798-2)
Supplement: Supplementary file 14 — Additional file 14. The key R script parameters of optimum beta value and co-expression matrix used for weighted gene co-expression network analysis. [file 12870_2020_2798_MOESM14_ESM.docx]

**Optimum beta value determined:**

powers = c(c(1:10), seq(from = 12, to=20, by=2))

sft = pickSoftThreshold(datExpr0, powerVector = powers, verbose = 5)

**Parameters used for constructing co-expression matrix:**

net = blockwiseModules(datExpr0,power = 10,maxBlockSize = 6000, TOMType = "unsigned", minModuleSize = 30, reassignThreshold = 0, mergeCutHeight = 0.25, numericLabels = TRUE, pamRespectsDendro = FALSE, saveTOMs = TRUE, saveTOMFileBase = "AS-green-FPKM-TOM", verbose = 3)
